# Separable Reversible Data Hiding in Encrypted Images for Remote Sensing Images

**DOI:** 10.3390/e25121632

**Published:** 2023-12-07

**Authors:** Ruihua Liu, Quan Zhou, Juanni Liu, Yi Zhang, Zheng Hui, Xuejiao Zhang

**Affiliations:** Xi’an Institute of Space Radio Technology, Xi’an 710000, China; lrh5042019@outlook.com (R.L.);

**Keywords:** reversible data hiding in encrypted images, high capacity, entropy retained, MSB compression, separability, remote sensing images

## Abstract

High security and effectiveness are critical performance metrics in the data transmission process for satellite remote sensing images, medical images, and so on. Previously, the receiver could gain a high-quality cover image (lossy) after decryption in a separable manner to balance embedding capacity (*EC*) and security. Completely separable, reversible data hiding in encrypted image (SRDH-EI) algorithms are proposed to address this issue. In this study, the cover image was preprocessed at the sender’s end. The pre-embedded pixels and most significant bits (MSB) were compressed via two coding methods to reserve space. Additionally, the header data were embedded for marking. Finally, auxiliary data and secret data were embedded in a forward “Z” and reverse “Z” shape before and after encryption, respectively. The receiver could extract secret data and decrypt the cover image separately using the keys and markers. The experimental results demonstrate that the algorithm reached a high *EC* for remote sensing images by utilizing pixel correlation at multiple positions within the groups. The cover image could maintain its entropy during the data embedding process, ensuring security. The decrypted image could be recovered without distortion, furthermore, the receiver could achieve complete separability, so it has good application prospects for remote sensing images.

## 1. Introduction

In traditional reversible data hiding (RDH), secret data can be embedded into a cover image to improve security. Embedding can be operated in the image spatial domain [1,2], transform domain [3,4], compressed domain [5,6], etc. The main characteristic is that the data are hidden and the content of the image is leaked. Therefore, the RDH in encrypted images (RDH-EI) has been developed and applied to natural images, medical images, cloud computing, and more. However, research on remote sensing images is limited. Some algorithms cannot recover the original image without distortion; yet, remote sensing images and the data they carry are very precious and cannot be destroyed. There is also a limitation in the embedding ability of traditional algorithms, so they have not been widely applied in remote sensing image applications. Actually, compared to other carriers such as natural images, most remote sensing images contain interesting objects as well as a large number of background regions, which is suitable for mining more space to embed data and beneficial for saving bandwidth resources. The data carried in remote sensing images are varied, such as image metadata (geographic location, date and time obtained, projection parameters, image resolution, etc.), satellite telemetry data, and some secret data, which can be synchronously transmitted to ensure real-time processing without increasing transmission resources. Some remote sensing images and data involve national defense security, military security, etc., and using RDHEI can protect them during transmission. Due to significant advantages in security and efficiency, it is a potential technology for remote sensing image transmission.

RDH-EI algorithms can usually be divided into two categories. One is a framework based on vacating a room after encryption (VRAE) [7,8,9,10,11,12,13,14,15,16,17,18,19,20] and the other is based on reserving a room before encryption (RRBE). Under the VRAE framework, the sender first encrypts the original cover image using encryption methods such as stream ciphers [7,8,9,10,11,12,13,14], block ciphers [15,16,17,18,19], and all homomorphic ciphers [20] and then designs hiding algorithms based on the different characteristics of the encrypted cover. Due to the loss of correlation between adjacent pixels after stream cipher encryption, Zhang [7] adopted a method of pixel bit flipping and wave function recovery for data embedding, extraction, and cover recovery. Other studies [8,9] have used the technique of compressing encrypted pixels to make room for embedding. Some studies [10,11,12] have designed transformation methods to provide space. In addition, block encryption preserves the correlation of intra-block pixels, which can be combined with traditional RDH techniques, such as histogram shift (HS), predicted difference encoding, and difference expansion in intra-block pixels [15,16,17,18,19], providing a new approach for improving the embedding capacity (*EC*) of the algorithm. Overall, due to the difficulty in mining the features of encrypted images, algorithms based on the VRAE framework have the following characteristics: low *EC*, low decryption quality of embedded pixels, and low complexity.

To improve the above issues, such as low *EC* and decryption quality, one study [21] proposed the RRBE frame. Other studies [22,23,24,25,26,27,28,29,30,31,32,33,34,35,36] based on the RRBE frame first analyzed and processed the original image to reserve space and then performed encryption and data embedding. For example, some studies [21,22] classified image blocks by calculating smoothing functions and designing thresholds, reserved the least significant bit (LSB) of the original block using RDH technology, and, finally, embedded data in the encrypted LSB position. In response to the demand for performance improvement, the solution focused on two aspects: (1) reserving space before encryption to increase embedding capacity and (2) combining preprocessing and data embedding to improve decryption quality. For (1), there are multiple methods, such as Huffman encoding [23], most significant bits (MSB) compression [24], and dynamic difference coding strategies for embedding objections [25,26]. For (2), some works [27,28,29,30,31] adopted high-precision prediction models, which improved the accuracy of predicted pixels [28,31] and combined prediction error correction to recover pixel values [27,29,30]. For example, one study [27] used predefined models for pixel classification, reserved space, and embedded data through predictive error correction, and finally restored pixels at the receiver through error correction data. Other research [32,33,34,35,36] adopted interpolation, permutation, quadratic processing, and other methods. For example, in one work [35], block permutation was used to scramble image data and then the pixels within the block were encrypted with the same stream cipher to ensure correlation. Finally, HS technology was used for data embedding to reduce modifications to pixel values. At the receiver’s end, the quality of the directly decrypted image was very close to the original cover image. In summary, under the RRBE framework, these algorithms have more significant potential in embedding capacity and decryption quality due to the full utilization of pixel correlation and the combination of traditional RDH techniques such as HS and differential expansion.

The above algorithms only included a single hider, which is currently the main research frame. However, in recent years, schemes [37,38] based on multiple hiders have also been developed, but the structure, processing flow, performance analysis, etc. are more complex. The research content of this paper is based on a single hider.

Two aspects need to be considered from the current research on information hiding in remote sensing images. Firstly, existing algorithms are divided into joint RDH-EI (JRDH-EI) and separable RDH-EI (SRDH-EI). At the receiver’s end, the cover image, metadata, and secret data are vital for remote sensing images. However, in most previous schemes, the decrypted image has been lossy, which may cause the loss of important data of remote sensing images at the receiver’s end. If lossless recovery is achieved by combining extracted data, complexity is increased and security is reduced. Thus, an SRDH-EI algorithm is more needed. Secondly, most previous schemes needed to balance *EC* and the decryption quality of the cover image. However, under the premise of SRDH-EI, the only focus is how to achieve high *EC*. 

In response to the above issues, this paper introduces RDH-EI technology into remote sensing images and proposes a high-capacity SRDH-EI algorithm based on the RRBE framework. This algorithm provides a theoretical basis and performance verification for applying RDH-EI technology to remote sensing images. In general, the main contributions are summarized as follows:

- An intra-block pixel classification compression is proposed that combines MSB compression with differential compression and, finally, reserves hidden space to improve transmission effectiveness;
- An entropy-retained method is proposed that ensures the encrypted cover image does not increase entropy after data embedding, thereby improving transmission security;
- A marker information embedding mode that combines a forward “*Z*” shape and a reverse “*Z*” shape is proposed before and after encryption, enabling the receiver to achieve complete separability, which is beneficial for the receiver to receive cover image data and secret data with different permissions, improving receiver flexibility and data security;
- There is no need for secret data extraction, and the decrypted cover image can be recovered without distortion. Thus, complete reversibility can be achieved.

The content of other chapters in this paper is arranged as follows. Section 2 introduces the related works. Section 3 provides a detailed explanation of the principles and steps of the proposed algorithm. Section 4 presents and discusses the performance of the algorithm and makes a comparison with other algorithms. Section 5 gives a summary and explores future research directions.

## 2. Related Works

Below are some related works from recent years. They involve MSB processing, data self-embedding, decryption of cover images for the receiver, etc.

### 2.1. MSB Processing

In most schemes, the map used to guide data embedding is usually embedded into the LSB bit of the encrypted cover image, resulting in the original LSB bit needing to be stored with the same length, such as in [26]. To reduce the cost of storing the original LSB bit, some works have also shifted the processing of the LSB to the MSB. The work [9] proposed a method to achieve high embedding payloads by combining MSB estimation with distributed source coding (DSC). Since estimating the MSB is much more accurate than estimating the LSB plane, achieving a large embedding capacity is possible. Therefore, considering the higher correlation of MSBs, the proposed algorithm places the marker position in the MSB and compresses the original MSB to save some space.

### 2.2. Data Self-Embedding

In most schemes, auxiliary and secret data are embedded into the cover image. Self-embedding refers to embedding a portion of data into its data during the preprocessing stage before encryption rather than during the data embedding stage. To reserve space, data self-embedding was performed on a cover image during the preprocessing step of work [27]. Before image encryption, error correction data were generated through preprocessing and reversibly embedded into the down-sampling pixels of the original cover image. After image encryption, only a few LSBs of each non-sampling pixel of groups needed to be retained or flipped to embed the data. However, this work produced low *EC*.

### 2.3. The Cover Image Decryption

In existing algorithms, the processing of the receiver is divided into separable and joint types. The joint algorithms must perform data extraction and cover decryption according to the designed order. To achieve true reversibility and separability, the two operations must be completed without errors, such as in [7,8,9,10,11,12,13,14,27,36], etc. Separable algorithms have more flexibility. That is, the cover image’s decryption and secret data extraction can be processed independently. This is very suitable for occasions with high confidentiality requirements, such as SRDH-EI works in [22,23,24,25,26,28,29,30,31,32,33,34,35], etc. In general, data extraction can be completed in both ways. The decrypted peak signal-to-noise ratio (PSNR) of the cover image can reach a high value. Works [27,28,29,30,31] improved the decryption quality by improving the accuracy of the prediction model and fully utilizing the correlation between the original cover image pixels. Work [33] proposed a progressive mechanism for the recovery image. The receiver divides the image into three pixel sets: square set, triangle set, and circle set, and the image is restored through three rounds of prediction. The iterations of the triangle and circle sets are carried out according to the prediction result of the square set. Therefore, the image presents the effect of progressive recovery.

Then, the predictions and iterations of the triangle and circle sets are carried out according to the result. Each round has its own predictive model.

According to the detailed analysis above, these methods have shown good performance in, for example, high *EC* and recovery quality. However, there are also some issues, such as the compression of auxiliary information location maps under the RRBE framework and the possibility of lossy decryption in SRDH-EI. This paper proposes new ideas to address these two issues.

## 3. Proposed Algorithm

**Figure 1:**
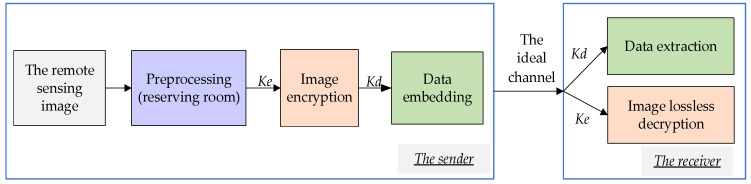
The framework of the proposed algorithm.

The proposed algorithm reserves space during the preprocessing stage through lossless compression encoding, utilizing the correlation of the cover image to improve the embedding capacity and decryption quality. Finally, the receiver can also recover the cover image losslessly. However, because the secret data embedding of the RDH-EI algorithm is completed in the encrypted image, which requires more encryption steps than traditional RDH, there are several points to note: (1) The hidden positions in the RDH algorithm will disappear after encryption. To ensure the accuracy of the hidden positions, it is necessary to mark the reserved hidden positions in the encrypted image. There will also be some auxiliary markers before encryption to achieve lossless recovery. (2) The hidden capacity will be changed, and the space vacated in the preprocessing stage needs to hide not only secret data but also auxiliary data. Therefore, exploring as much space as possible and reducing the amount of auxiliary data are necessary. The framework of the algorithm is shown in Figure 1.

As shown in Figure 1, the process contains two parts: the sender and the receiver. The function of the sender is to hide the secret input data in the cover image and transmit them. The sender first preprocesses the image to reserve space before encryption. Then, the encryption key *Ke* is used to encrypt the marked image to obtain the encrypted image. Finally, the secret data are embedded into the encrypted image by the hidden key *Kd* and secondary labeling is conducted. At this point, the sender transmits the image containing the secret data. It is assumed that the image is transmitted in an ideal channel without noise interference, so the received image is not distorted. The function of the receiver is to recover secret data and the cover image correctly. It can access the corresponding data based on the type of key they have. Separability can be achieved by the proposed algorithm.

### 3.1. Processing Steps for the Sender

#### 3.1.1. Preprocessing

**Figure 2:**
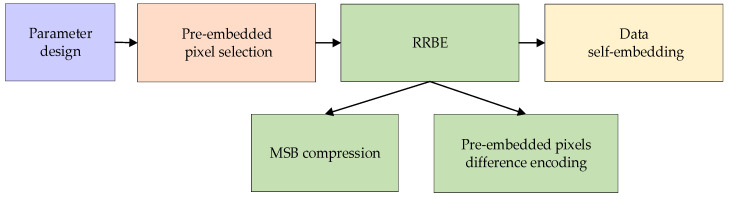
The steps of preprocessing.

Figure 2 shows the main steps of preprocessing. This process includes parameter design, pre-embedded pixel selecting, auxiliary data encoding, MSB compression, data self-embedding, and initial labeling to obtain the marked image. The specific steps are described as follows:

*Step 1*: Block the cover image ***I*** with a size of *M* × *N* to obtain blocks with a size of *s* × *s*. This processing step is conducive to evenly distributing the embedded data in the cover image, which can reduce the probability of continuous error codes of secret data during transmission.

*Step 2*: Divide blocks into groups. To fully utilize pixel correlation while ensuring the recovery quality of the image, every 4 pixels are grouped as {*x*_1_, *x*_2_, *x*_3_, *x*_4_}, and there are a maximum of *t* groups within each block, where *t* = (*s* × *s*)/4. Set parameter *β* represents the number of hidden groups within the block, then *β* ≤ *t*.

*Step 3*: Design the parameter *α*, which is used to specify the positions that can be hidden in a group, with multiple positions used to provide greater embedding capacity. The specific rules are as follows:

(1) $$\alpha =\left\{\begin{array}{l} 1\;x_{2}\\ 2\;x_{2},x_{3}\\ 3\;x_{2},x_{3},x_{4} \end{array}\right.$$

*Step 4*: Calculate the difference of adjacent pixels within the block, and the difference ***d*** between the three positions is as follows:

(2) $$d=\left\{d_{2},d_{3},d_{4}\right\}=\left\{x_{2}-x_{1},x_{3}-x_{2},x_{4}-x_{3}\right\}$$

**Figure 3:**
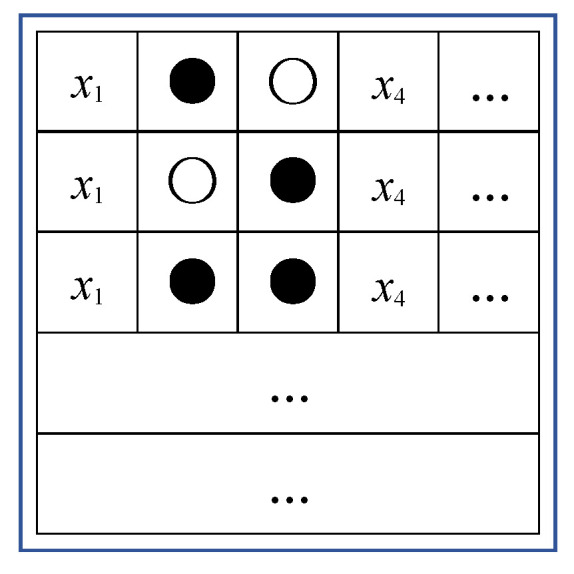
Pixel classification.

*Step 5*: Design threshold parameter *T* for pixel classification. The value is as follows:

(3) $$T=2^{n}-1$$

where *n* > 0, which represents the number of bits. In step 4, classify the pixels corresponding to the position where the absolute value of ***d*** is not greater than *T* as pre-embedded pixels. Therefore, all pixels can be classified as pre-embedded (PE) pixels and non-pre-embedded (NPE) pixels. In Figure 3, the symbol “●” indicates that the pixel at that position is a PE pixel that can be used for data embedding, while the symbol “○” indicates the opposite. As shown in Figure 3, assuming that *α* = 2, *β* = 3, the positions used for hiding in the group are *x*_2_ and *x*_3_, with three groups participating in hiding in each block. 

Next, generate a classification position map based on the above pixel classification results, record the pixel categories, and use this to guide subsequent processing. In the above steps, set the position corresponding to “●” in the position map to 1; otherwise, set it to 0. The size of a position map is (*M* × *N*)/(*s* × *s*) × *β*, and there is a total of α such maps. For example, in the case shown in Figure 3, the first position map represents whether *x*_2_ in each group is a PE pixel, with values in the position map being 101…; the second position map represents whether *x*_3_ in each group is a PE pixel, with values in the position map being 011…. These position maps are called *Maps data.*

*Step 6*: Encode the difference corresponding to the positions of the pre-embedded pixels mentioned above. The encoding is presented in binary form, consisting of positive or negative and absolute differences. The positivity and negativity are represented by 1 bit, with bit “1” representing a value not smaller than 0. Otherwise, the value of 1 bit is “0”. According to *T* in step 5, it can be determined that the encoding of the absolute value of the difference needs *n* bits. Therefore, the encoding difference corresponding to a single pre-embedded pixel is *n_f_* bits, where *n_f_* = *n* + 1, and the *n_f_* value cannot exceed 9 in a gray image (the max pixel value is not bigger than 255). Then, all the encoding is arranged in order in the position map to form the compressed difference code stream data *CS*_1_ with a length of *L_CS_*_1_, which is used for data recovery at the receiver’s end.

*Step* 7: Compress the MSB of *x*_1_. When *α* and *β* are set, the groups involved in processing are also determined. Extract original *m* bits named MSB*m* of *x*_1_ in each group, then compress the MSB*m* using run-length encoding to obtain the compressed MSB*m* stream *CS*_2_ with a length of *L_CS_*_2_. In the meantime, store MSBs of NPE pixels as *MSB0* with a length of *L_MSB0_*.

**Figure 4:**
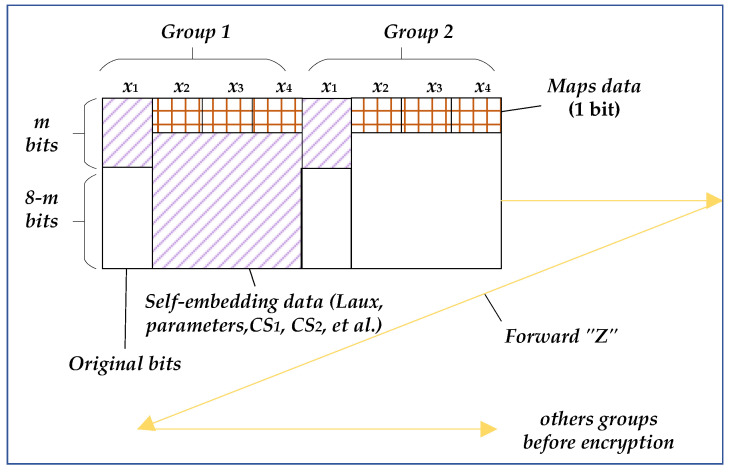
Auxiliary data self-embedding before encryption.

*Step* 8: Initial labeling of auxiliary data and data self-embedding. The embedded schematic diagram is shown in Figure 4. The two groups demonstrated represent different types. The first group means that the pixels all belong to PE pixels so that data can be embedded in the *m* bits of *x*_1_ and the 7 bits of PE pixels. The second group indicates that all the pixels belong to NPE pixels, which still consist of the original 7 bits and cannot be embedded data. However, the data at these two positions in all groups have changed. One is the *m* bits of *x*_1_ involved in compression and the other is that MSBs must be marked.

The auxiliary data self-embedding rules designed for this stage are as follows:

(a) Auxiliary data. This includes the header data (total length of auxiliary data *L_aux_*, parameters *α* (2 bits), *β* (4 bits), *m* (3 bits), *n_f_* (4 bits)), *CS*_1_, *CS*_2_, *MSB0*, and partial *Maps data*.
(b) Embedding direction and position. The position follows the forward “*Z*” shape, starting from the top lefthand corner. Embed *m* MSB bits in *x*_1_ and lower 7 bits in PE pixels for each group. Because the lower 7 bits in PE pixels are fixed, *L_aux_*, *α*, *β*, *m*, and *n_f_* must be embedded in the frontmost *x*_2_ (PE pixels).
(c) Embedding process. Firstly, according to *α* and *β*, embed *Maps data map*_1_, *map*_2_, and *map*_3_ into the MSB of *x*_2_–*x*_4_. Then, for *x*_1_, directly embed *m* bits and then judge the MSB of *x*_2_–*x*_4_. If it is 1, embed the 7-bit data; otherwise, skip the pixel. In this way, all auxiliary data mentioned in step (a) are embedded into the cover image before encryption.

After the above preprocessing steps, the marked image with reserved space is obtained.

#### 3.1.2. Encryption

Encrypt the marked image using encryption key *Ke*. Assuming that the range of pixel grayscale values *f* (*i*, *j*) at the position (*i*, *j*) in the marked image is [0, 255]. Each pixel can be represented as bits *b_i_*_,*j*,*k*_, with *k* values [1, 8]. The relationship between the grayscale values *f* (*i*, *j*) and *b_i_*_,*j*,*k*_ is as follows:

(4) $$b_{i,j,k}=\left\lfloor \frac{f(i,j)}{2^{k-1}}\right\rfloor \mathrm{mod}2,\;k=1,2,\ldots 8$$

(5) $$f(i,j)=\sum_{k=1}^{8}(b_{i,j,k}\times 2^{k-1}),\;k=1,2,\ldots 8$$

Then, use *Ke* to generate a pseudo-random binary array *r_i_*_,*j*,*k*_, and perform an XOR operation with *b_i_*_,*j*,*k*_. The calculation is as follows:

(6) $$B_{i,j,k}=b_{i,j,k}⊕r_{i,j,k}$$

where *B_i_*_,*j*,*k*_ is the results in encrypted bit form. The encryption key *Ke* also serves as the decryption key and has reversibility, ensuring complete restoration of the image content before encryption during the decryption phase. Through this step, the encrypted image can be obtained, and the content of the cover image is protected.

#### 3.1.3. Data Embedding

**Figure 5:**
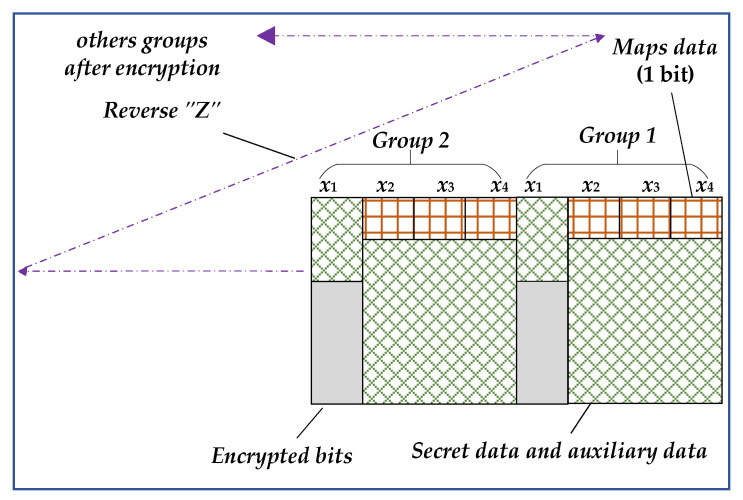
Secret data embedding after encryption.

The function of this section is to embed secret data in the encrypted image. The processing process is shown in Figure 5, and the pixels in the two groups shown here belong to PE pixels.

The secret data embedding rules designed for this stage are as follows:

(a) The data to be embedded. They contain the header data (total length *L*, parameters *α*, *β*, *m*), the remaining *Maps data*, and secret data *S*.
(b) Embedding direction and position. Because the self-embedding of auxiliary data has been completed in the preprocessing stage, and some PE pixels have been used, a reverse “*Z*” direction processing is adopted to facilitate extraction, as shown in Figure 5. This section starts from the bottom righthand corner of the encrypted image based on the remaining *Maps data*. Embed *m* MSB bits in *x*_1_ and the lower 7 bits in PE pixels. The pixels in the two groups shown in Figure 5 belong to PE pixels, so both are embedded with the data. Similarly, *L*, *α*, *β,* and *m* must be embedded in *x*_2_ (PE).
(c) Embedding process. This is similar to the self-embedding process. According to *α* and *β*, embed the remaining *Maps data* into the MSB bits of encrypted pixels. Then, for *x*_1_, directly embed *m* MSB bits and then judge the MSB of *x*_2_–*x*_4_. If it is 1, embed the data; otherwise, skip the pixel.

Compared to the self-embedding process in Section 3.1.1, there are three differences: (1) The length of the embedded data is different in header data. *L_aux_* and *L* need to be calculated. (2) The content of the parameters in the header data is different. The parameter *n_f_* is embedded before encryption and used to decompress the stream *CS*_1_ at the receiver. However, the secret data to be embedded after encryption do not require this parameter. (3) The direction of twice data embedding is different. The direction of the auxiliary data self-embedding is a forward “*Z*” shape, while it is a reverse “*Z*” shape for the latter secret data embedding process.

In this step, the transmitted image containing secret data for the sender processes is obtained. Embedding auxiliary data before encryption and secret data after encryption is beneficial for the receiver to process data in a separable manner. Subsequently, the image containing secret data is transmitted from the sender to the receiver through an ideal channel, during which neither type of data is subjected to noise attacks.

### 3.2. Processing Steps for the Receiver

#### 3.2.1. The Receiver Only Owns the Hidden Key *Kd*

This process is the inverse of the embedding process shown in Figure 5. The received image is divided into blocks with the size of *s* ×*s* and then processed in groups of every 4 pixels. The data extraction position starts from the bottom righthand corner of the cover image and is processed in the direction of a reverse “*Z*” shape. The secret data extraction rules are as follows:

(a) According to the MSB marker (*Maps data*) of *x*_2_, the header data (*L*, *α*, *β, m*) can be extracted from the PE pixels of *x*_2_. Then, based on these data, the length of data to be extracted, the positions of groups involved, and the pixels can be determined. Here, the header data with a fixed length are extracted from the *x*_2_ (when MSB of *x*_2_ is 1) of the first group in each block.
(b) Based on the specific groups and pixel positions determined in (a), by judging the MSB marker, the pixels are divided into PE pixels and NPE pixels. Then, extract all secret data from low 7 bits of PE pixels and the high *m* MSB bits of *x*_1_ in the corresponding groups. According to the rules designed by the sender, except for fixed header data, the remaining data are secret data.

Note that the receiver can only extract secret data, and the content of the cover image is unknown, so it is protected.

#### 3.2.2. The Receiver Only Owns the Key *Ke*

This process is the inverse of the embedding process shown in Figure 4. The cover image decryption and recovery rules are as follows:

(a) The key *Ke* is used to directly decrypt the received image to obtain the preliminary decrypted image *De*_1_. Because the decryption operation is reversible, only the pixels embedded in the data are distorted, and other pixels can be completely restored.
(b) Then, process *De*_1_ in groups of 4 pixels. The process of extracting data is the same as in Section 3.2.1, where auxiliary data are sequentially extracted. According to the formatting of the embedded data in Figure 4, the extracted data are classified into the header data (the length data *L_aux_* and parameters (*α*, *β*, *m*, *n_f_*)) and other stream data (*CS*_1_, *CS*_2_, *MSB0*).
(c) Recover original pixels using auxiliary data based on *De*_1_. For PE pixels and *x*_1_, firstly, decompress the MSB stream *CS*_2_ through run length decoding to obtain the original MSB*m* data, and replace high *m* bits of *x*_1_ with MSB*m* to recover *x*_1_; then, based on the parameter *n_f_*, the difference compressed bitstream *CS*_1_ is decompressed and restored; next, combine *x*_1_ and *CS*_1_. Other pixels *x*_2_–*x*_4_ belonging to PE pixels within the group can be restored using the inverse process of Formula (2). For NPE pixels, use *MSB0* to replace the MSB of NPE pixels to restore original values. This stage is symmetrical with the preprocessing stage, so the pixels are completely reversible without distortion.

At this point, the receiver obtains the final decrypted image, *De*_2_, which is a completely reversible image. At the same time, this stage only extracts auxiliary data for the cover image recovery, so the secret data are protected.

From Section 3.1.1 and Section 3.1.2, it can be found that it is not necessary to own two keys, *Kd* and *Ke*, simultaneously to obtain the corresponding data entirely separately. In previous algorithms, in most cases, the decrypted image was lossy, and only by possessing both types of keys could a lossless cover be obtained. Therefore, the proposed algorithm has the characteristics of complete separability and true reversibility.

### 3.3. An Example of the Proposed Algorithm

**Figure 6:**
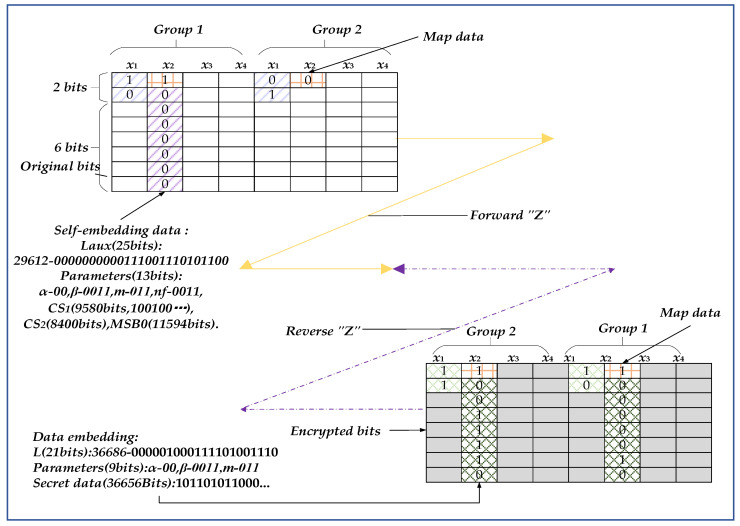
An example of the proposed algorithm.

**Figure 7:**
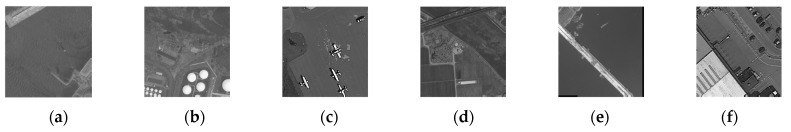
The remote sensing images. Ships and sea areas (**a**,**d**,**e**); land (**b**); airports (**c**); and parking lots (**f**).

Figure 6 demonstrates the process of the algorithm more clearly, taking Figure 7a as an example, the sender and receiver are explained separately. Here, *M* = *N* = 512 and *s* = 8.

(a) Processing steps for the sender

Firstly, assume that *α* = 1, *β* = 4, *m* = 2, and *n_f_* = 4. Parameters represent that the first four groups (*β* = 4) within a block participate in the operation; the high 2 MSB bits (*m* = 2) of *x*_1_ are compressed in a group; only *x*_2_ (*α* = 1) participates in difference compression and hiding; every 4 bits (*n_f_* = 4) signifies a difference in coding. 

Then, calculate and store the auxiliary data *CS*_1_, *CS*_2_, and *MSB0* for recovery. Taking the above parameters as an example, the results of Figure 7a are as follows. Considering the extreme case, *L_aux_* is represented by 25 bits, parameters are 13 bits, *L_CS_*_1_ is 9580 bits, *L_CS_*_2_ is 8400 bits, *L_MSB_*_0_ is 11,594 bits, the number of PE pixels is 4790, and the number of NPE pixels is 11,594. The length of self-embedded data before encryption is as follows:

(7) $$L_{aux}=L_{CS_{1}}+L_{CS_{2}}+L_{MSB0}+25+13=29,612(\mathrm{bits})$$

Next, arrange the above data into a fixed format bitstream, including header data (*α*, *β*, *m,* and *n_f_*) and auxiliary data (*CS*_1_, *CS*_2_, and *MSB0*) and then mark the *Map data* according to the upper lefthand corner of Figure 6. According to the markers, self-embed the above bitstream data (29,612 bits in total). Due to *α* = 1, only MSB bits of *x*_2_ are embedded in the *Map data*, and then, based on the *Map data*, 25 bits of *L_aux_* and 13 bits of parameters are embedded in PE pixels (38 bits in total). Starting from the first group again, embed data in the high 2 bits of *x*_1_ and the low 7 bits of PE pixels (skipping the first 38 bits in PE pixels). After self-embedding all auxiliary data in the forward “*Z*” shape, the marked image is obtained and then encrypted by *Ke* to generate the encrypted image.

The embedded data after encryption include header data (total length data *L*, parameters (*α*, *β,* and *m*)) and secret data. Under the above parameters, *L* is represented by 21 bits, and parameters are 9 bits. The total length of encrypted data embedding is as follows:

(8) $$L=4790\times 7+(M\times N)/(s\times s)\times m\times \beta -L_{aux}=36,686(\mathrm{bits})$$

The capacity of secret data is as follows:

(9) EC=L−(21+9)=36,656(bits)

Finally, convert the above data and parameters into binaries. The header data and secret data are embedded into the encrypted image in the reverse “Z” shape, similar to the principle of embedding before encryption, as shown in the bottom righthand corner of Figure 6. Firstly, embed the remaining *Map data*, then embed header data (21 bits of *L* and parameters of 9 bits, 30 bits in total) in the PE pixels according to the instructions of MSB. At last, embed secret data (36,656 bits) in the remaining positions of PE pixels to obtain the transmitted image.

(b) Processing steps for the receiver

If secret data need to be extracted, the operation will start from the bottom righthand corner of the transmitted image and follow the reverse “Z” shape, as shown in the bottom righthand corner of Figure 6. Firstly, block and group the transmitted image and judge whether the MSB at *x*_2_ is 1. If it is 1, extract the lower 7 bits until 30 bits are extracted and pause. Then, divide 30 bits into length *L* of 21 bits and parameters of 9 bits (*α*, *β,* and *m*). Determine all the groups involved in hiding based on the parameters and then extract all secret data from the high 2 bits of *x*_1_ and the low 7 bits of PE pixels. 

To obtain the decrypted image, directly decrypt it with *Ke* to obtain *De*_1_ and then start from the top lefthand corner of the transmitted image and process it in a forward “*Z*” shape. Consistent with the process of extracting secret data mentioned above, first extract the length data as 25 bits of *L_aux_* and 13 bits of parameters (*α*, *β*, *m,* and *n_f_*). Then, extract other auxiliary data based on the above parameters. According to the arrangement rules of the sender, several types of data are reclassified to obtain auxiliary data (*CS*_1_, *CS*_2_, and *MSB0*). Finally, follow the processes shown in Section 3.1.1 and Section 3.2.2 to perform bitstream decompression and inverse processing. Ultimately, all pixels are recovered losslessly.

The above example shows the detailed process of the algorithm. The process and experimental results are similar for selecting other parameters or test images.

## 4. Experimental Results and Discussion

This experiment selected some images to analyze the relevant parameters and performance of the algorithm. Firstly, six images were randomly chosen from the DOTA remote sensing image dataset [39] and processed into 512 × 512 × 8 grayscale images for simulation. Then, to compare the performance with relevant algorithms, simulations were conducted on other test datasets, and representative images of texture smoothing and complexity were selected for detailed analysis and comparison with relevant algorithms. The operation system simulated in this experiment was Windows 10 and the processor was Inter (R) Core (TM) i7-7700 CPU @ 3.6 GHZ 3.6 GHZ.

**Figure 8:**
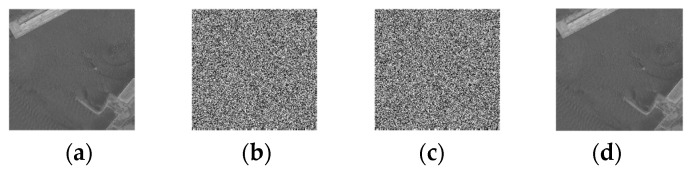
Process diagrams of Figure 7a at each stage. (**a**) The cover image; (**b**) The encrypted image; (**c**) The transmitted image;(**d**) The decrypted image.

Figure 7 shows the remote sensing test images, including scenes such as ships and sea areas (a), (d), and (e); land (b); airports (c); and parking lots (f). Figure 7 shows the process images of Figure 7a at each algorithm step. Figure 8a–d show the original image, the encrypted image, the secret image (the transmitted image), and the decrypted image in the sequence. From a security perspective, the encrypted image and the secret image were visually imperceptible, safeguarding the cover image and secret data. At the same time, subfigure (d) was visually identical to the cover image (a) after decryption and recovery.

In the experiment, parameters *M* and *N* and the block size *s* × *s* affected the secret data distribution but did not affect *EC* and decryption performance. Here, *M* × *N* = 512 × 512 and *s* × *s*= 8 × 8. They were used as an example for the experiment. Thus, *t* = 8 × 8/4 =16 indicated up to 16 available groups in an image block, that is, *β ≤ 16*. The experiment mainly studied the impact of four parameters: *α*, *β, m,* and *T (or n_f_*) on algorithm performance. 

Regarding the security of transmission, the changes in entropy are analyzed in Section 4.1. For the effectiveness of transmission, *EC* is simulated and studied in Section 4.2. The recovery quality of the cover image is analyzed in Section 4.3. Then, in Section 4.4, the other two test datasets are simulated and compared with other algorithms. Finally, in Section 4.5, the algorithm proposed is discussed and analyzed.

### 4.1. Entropy

Entropy reflects the amount of information in an image, and its calculation is as follows:

(10) $$H(s)=-\sum_{i=0}^{2^{n}-1}p(s_{i})\times \log_{2}(p(s_{i}))$$

where *P*(*s_i_*) represents the proportion of pixels with a grayscale value of *s_i_* in an image, *n* represents the number of grayscale levels, and, for grayscale images, *n* = 8. For each stage of the algorithm, if the entropy fluctuates little, there is less change in the amount of information. If the entropy changes significantly, this means that the image is more likely to be detected for changes. In the experiment, *EC* significantly impacted entropy fluctuation, while among the parameters that affected the capacity, the influence of *m* was relatively small. Therefore, it was assumed that *m* = 0. *T* = 15, which meant that *n_f_* = 5.

Table 1 shows the entropy values and changes in the image at each stage. The original entropy is displayed in the first column. Columns 2 and 3 show the entropy after encryption and the change between it and the original entropy (Change 1). It can be seen that after encryption, the information entropy of the image tended to be 7.99, which is a relatively stable value because random sequences generate stream ciphers. This phenomenon also indicates that the information entropy of the encrypted image increased and the content inside the image tended to be chaotic and disorderly, resulting in improved security. Columns 4 and 5 in Table 1 provide the entropy after embedding the data in the encrypted image and the changes between it and the original entropy (Change 2). By comparison, it can be found that Change 2 was close to Change 1, indicating that the image was still secure after embedding the data. Specific data are provided in column 6 (Change 3) to describe the change before and after data embedding accurately. It can be seen that when *α* = 3, *β* = 8, and *T* = 15, the value of Change 3 was around 0–0.02, indicating that entropy remained stable and the fluctuation range was very small.

**Figure 9:**
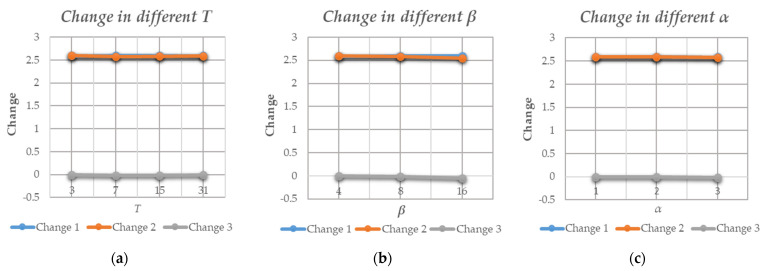
Entropy changes in different parameters. (**a**) Parameter *T*; (**b**) Parameter *β*; (**c**) Parameter *α*.

Figure 9 shows the influence of three parameters on entropy and provides corresponding variation values. Among them, the values of *T* are 3, 7, 15, and 31, seen in Figure 9a; the values of *β* are 4, 8, and 16, seen in Figure 9b; and the values of α are 1, 2, and 3, seen in Figure 9c. The curves in the figures show that Changes 1 and 2 were always close and stable and Change 3 was fluctuating around 0. This case was consistent with the phenomenon in Table 1, indicating that entropy values remained stable under different parameter combinations.

From the above data, it can be seen that in the two stages of encryption and data embedding, the entropy value tended to be the entropy value of the noisy image and remained almost unchanged, which reflects that there was no increase in the information amount of the cover image during the transmission process, which is conducive to transmission security. This feature was also mentioned in works [12,13], which studied the stable entropy of encrypted images, reflecting the security of the algorithm. Therefore, the proposed algorithm also possesses this property and security.

### 4.2. Embedding Capacity

The *EC* reflects the pure embedding capacity of the cover image, expressed in bits per pixel (bpp). In the proposed algorithm, *EC* was mainly determined by the following factors: (1) The space *EC*_1_ compressed by the MSB of *x*_1_ (determined by *β* and *m*, the cost is *L_CS_*_2_). (2) The space *EC*_2_ brought by PE pixels (determined by *α*, *β,* and *T*, the cost includes 68 bits of twice header data and auxiliary data *L_CS_*_1_, *L_CS_*_2_, and *L_MSB0_*). The *EC* of the cover image was calculated according to the following formulas:

(11) $$EC=EC_{1}+EC_{2}$$

(12) $$EC_{1}=((M\times N)/(s\times s)\times \beta \times m-L_{CS_{2}})/(M\times N)$$

(13) $$EC_{2}=((M\times N)/(s\times s)\times \alpha \times \beta -L_{CS_{1}}-L_{MSB0}-68)/(M\times N)$$

Table 2 shows the experimental results of *EC*_1_. In theory, in the preprocessing stage of the original image, the correlation of MSB*m* was very strong, so it provided more compression space. But as *m* increased, the correlation between bit layers weakened, and the compression effect gradually decreased. From the table, it can be seen that for Figure 7a, when *m* = 2, that is, the highest and second-highest bits of the pixel were used for compression, the compressed space was higher than in other cases. When the compressed bit layer, *m,* was determined, more bits participated in compression (increasing *β*), and there was more available space, *EC*_1_. In the table, the maximum *EC*_1_ is 0.407 bpp. The maximum values in Table 2, Table 3, Table 4, Table 5 and Table 6 are marked in bold.

*EC*_2_ was influenced by multiple factors (*α*, *β*, *T,* and the cost), and the final *EC* mainly depended on *EC*_2_. Therefore, the combined effect of *EC*_2_ and *EC*_2_ on *EC* is shown in the following tables. Here, assume that *m* has already reached the optimal value (*m* = 2 for Figure 7a). Table 3, Table 4 and Table 5 show the experimental results of the algorithm in Figure 7a. Table 6 shows the experimental results of the algorithm in Figure 7b. The experimental results are those of *EC* (bpp), decryption quality (dB), and structure similarity index measure (SSIM). 

As shown in Table 3, Table 4 and Table 5, the experimental results of three parameters under different conditions are presented. Table 3 studies the impact of parameter *α* on *EC*. The other two parameters remained unchanged, assuming *β* = 4 and *T* = 1. According to the number of hidden positions in the grouping during algorithm design, *α* took values of 1, 2, and 3, respectively. Table 3 shows that the *EC* ranged from 0.140 bpp to 0.234 bpp. Besides the capacity of 0.09 bpp by MSB compression when *β* = 4, the *EC* at all three positions were 0.050 bpp, 0.053 bpp, and 0.047 bpp, respectively, indicating that the embedding ability of the three positions was similar.

The influence of parameter *β* on embedding capacity is studied in Table 4, assuming *T* = 1 and *α* = 3. According to the size of image segmentation and grouping, it can be seen that *β* ≤ *t* = 16, indicating that up to 16 groups in a block could be used for hiding. Here, 5 of these sets are used as examples to illustrate the situation. The experimental data show that *EC* ranged from 0.051 bpp to 0.969 bpp when *β* was from 1 to 16, meaning that the maximum *EC* was 0.969 bpp when all three positions in each group participated in hiding, and the difference was not greater than 1. At the same time, the larger *β*, the greater the embedding capacity.

Table 5 and Table 6 show the effect of parameter *T* on *EC*, where *α* = 3 and *β* = 16. *T* values were 1, 3, 7, and 15. Table 5 shows that the *EC* achieved its maximum value at *T* = *7* (2.234 bpp) for Figure 7a but at *T* = 3 (2.428 bpp) for Figure 7b. This indicates that there was not a linear relationship between the *EC* and *T*, and there was a certain *T* that maximized the *EC*. Therefore, among the three parameters, the *EC* increased with the increase of *α* and *β*, but only when *T* took an appropriate value could the optimal *EC* be achieved. Thus, the maximum *EC* is listed in Table 7, and the data display that the *EC* was relatively large, with an average of around 2.40 bpp.

### 4.3. Decryption Quality

Figure 1 shows only two types of operations at the receiver. Specific processing methods for two cases are illustrated in Section 3.2. In theory, the proposed algorithm could achieve complete separability. That is, a lossless cover image could be obtained by decrypting the received image and recovering the decrypted image without *Kd*.

Below is an analysis of the decryption quality. In Table 3, Table 4, Table 5 and Table 6, the experimental data show that the PSNR of the decrypted cover image was infinite, and the SSIM value was 1. They indicate that the decrypted and processed cover image was consistent with the original image, achieving the performance of distortion-free recovery. Moreover, all secret data in the experiment could be accurately extracted and recovered without error. Therefore, the proposed algorithm could be successfully implemented by the receiver in a separable manner designed for the sender. This is also an advantage compared to other algorithms. For example, in works [11,15,22,24,25,26,31,33], etc., three data recovery methods were generally designed for the receiver based on the type of keys they own. Only lossy cover images or secret data can be obtained if *Ke* or *Kd* is owned separately. Only when two kinds of keys are owned can the cover image be recovered without distortion. However, in the proposed algorithm, there are only two options for the receiver, that is, *Kd* or *Ke*, owned separately. The secret data and the cover image can be obtained without distortion in these two cases. That is, the PSNR of the decrypted image reaches infinity. The experimental data in Table 3, Table 4, Table 5 and Table 6 are consistent with the theoretical analysis, meaning the algorithm is completely separable and more flexible.

The above analysis and the transmission process shown in Figure 8 demonstrate that the proposed algorithm can ensure a high *EC* of secret data to improve effectiveness, achieve lossless decryption and error-free secure data transmission, and provide security for remote sensing images.

### 4.4. Tests on Other Datasets

**Figure 10:**
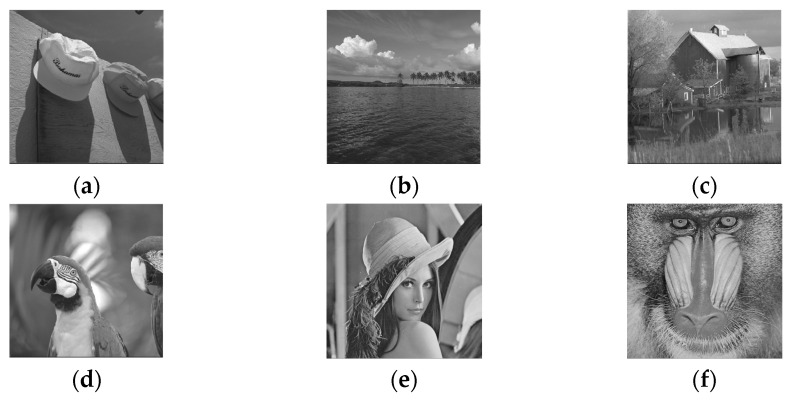
Tests on other datasets. (**a**) The ball caps; (**b**) The island; (**c**) The barn; (**d**) The birds; (**e**) Lena; (**f**) Baboon.

The proposed algorithm was tested on two other datasets. Test images are shown in Figure 10. There are four natural test images [40] from the Kodak dataset and two standard test images [41] from the University of Granada for simulation analysis and comparison with relevant algorithms.

Table 8 lists the test results on the two datasets in Figure 10. The experimental results show that the average *EC* was 1.74 bpp. Images (c) and (f) contained more textures, and the *EC* was lower than that for other images. For most natural images, including (a), (b), and (d) in the Kodak dataset, the *EC* was higher than that for other test images in the dataset [41].

**Figure 11:**
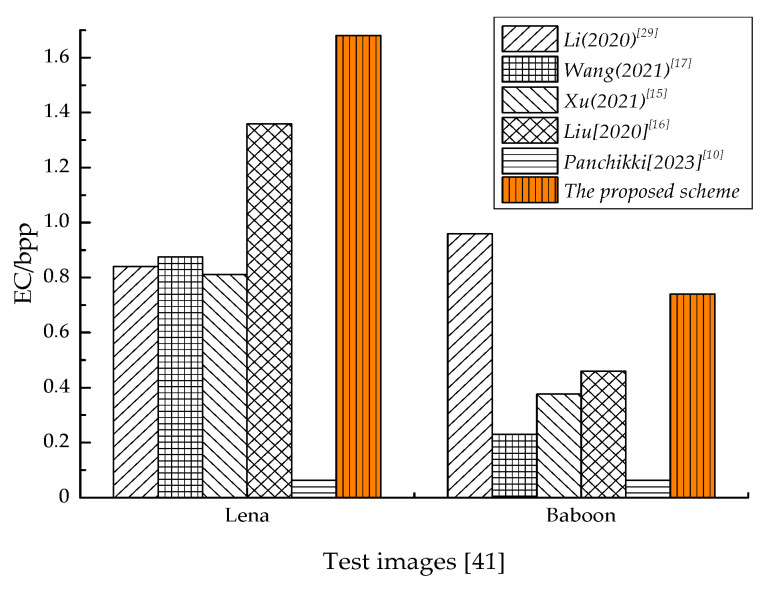
Comparison of embedding capacity.

Figure 11 shows the analysis and comparison of the proposed algorithm and several high *EC* algorithms in terms of maximum embedding capacity. As shown in Figure 11, for smooth images such as Lena, the *EC* was better than that for other algorithms, but for complex images such as Baboon, the *EC* was lower than *Li* [29] but also better than that for other algorithms. Because the proposed algorithm relies on the grayscale distribution characteristics of the image itself, images with complex textures had uneven distribution, resulting in less available space and affecting the *EC*. Overall, the proposed algorithm processed the three adjacent available positions in the group, resulting in the high utilization of the participating positions. Therefore, the embedding ability has excellent advantages.

**Figure 12:**
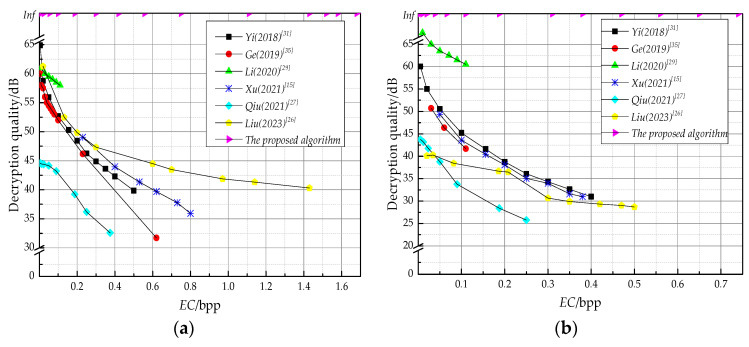
Comparison of decryption performance. (**a**) Lena; (**b**) Baboon.

Although the *EC* varied across datasets, the performance in decryption recovery was consistent. PSNR can be obtained from Table 3, Table 4, Table 5 and Table 6. Within the maximum *EC*, the PSNR values after decryption was infinite. Thus, the relationship between PSNR and *EC* was no longer drawn one by one, and they were consistent with the regularities shown in Figure 12. 

Figure 12 gives a comparative analysis of two standard test images under different algorithms. The horizontal axis represents the *EC* (bpp) and the vertical axis represents the decryption quality (dB). Firstly, as shown by the curves, within the maximum *EC* of Lena, the decryption quality of other algorithms remained above 30 dB, with good decryption performance. However, the decryption quality of the proposed algorithm is infinite, so the decryption performance is superior to that of other algorithms. The same conclusion could be obtained from the Baboon image, but the PSNR was lower than 30 dB at the maximum *EC* in some works [26,27]. Moreover, the *EC* of these schemes was lower than that of the proposed algorithm. Therefore, compared with other algorithms, the proposed algorithm’s performance has advantages in both *EC* and decryption quality. 

Table 9 compares the metrics with other related works. In the studies mentioned above, works [26,28,30,36] adopted the framework of RRBE, which can fully utilize the correlation within an original image to improve its *EC*, image recovery quality, and other performances. With SRDH-EI works designed in [9,22,26,28,30], the receiver could obtain the data according to requirements and permissions, resulting in higher security and flexibility. In the above works, the image the receiver obtained was lossy when possessing the decryption key. Therefore, it was necessary to have the hidden key to restore the cover image without distortion. In the proposed algorithm, the cover image can be restored without distortion when only the decryption key is owned. This is because auxiliary data self-embedding is performed before encryption during separability design, which has the advantage of completely separating the recovery of the cover image and the extraction of secret data. Work [27] also adopted the idea of data self-embedding before encryption. But the *EC* was low and the decrypted image was lossy. Therefore, compared to related works, the proposed algorithm can achieve true separability, making the processing of the receiver more flexible and secure.

### 4.5. Discussion

In Section 4.1, Section 4.2, Section 4.3 and Section 4.4, we demonstrated and analyzed the process and performance of the proposed algorithm.

For security, in Section 4.1, the entropy of the cover image before and after encryption and embedding was detected and analyzed in Table 1 and Figure 9. The entropy value could be retained during encryption and subsequent processing, and the transmitted image did not have any visual differences. This means that the cover image was secure during transmission and the content could be protected. At the same time, secret data were hidden within the encrypted image, making them invisible and safe.

For effectiveness, the pure load capability of the proposed algorithm was tested in Section 4.2. Table 2, Table 3, Table 4, Table 5, Table 6 and Table 7 show the experimental results and maximum embedding ability under different parameters. The experimental data demonstrate that the proposed algorithm could achieve an average *EC* of 2.40 bpp on remote sensing images. Experiments were conducted on other datasets in Section 4.4 for comparison. Compared with other works, the *EC* still has advantages.

For separability, the proposed algorithm was described in Section 3. The operation of the receiver was guided by embedding parameters twice. At the same time, the self-embedding of auxiliary data was introduced before encryption according to the forward “Z” shape and secret data were embedded according to the reverse “*Z*” shape to achieve separability. From the experimental data in Section 4.2 and the analysis in Section 4.3, this effectively achieved separability and ensured complete reversibility of the cover image and secret data.

Through the above analysis, we can see that the proposed algorithm has excellent performance. However, some points can be noted. In the example in Section 3.3, the actual lengths of various types of data are given. In the two embeddings before and after encryption, the length of secret data, i.e., pure capacity (*EC*), accounted for 55.29%, and the auxiliary data accounted for 44.61%. This indicates that the hidden space of the algorithm was very large, but nearly half of the space was used by auxiliary data. The utilization rate of the hidden space of the proposed algorithm was lower than 100% of schemes [7,27] without auxiliary data. However, due to the presence of auxiliary data, the proposed algorithm could achieve lossless recovery with only the key *Ke*. Other schemes, such as [7,26,27,33], require certain special conditions to be met in order to achieve lossless image recovery.

In summary, compared with the RDH-EI mentioned above, the proposed algorithm has the advantages of significant security, effectiveness, and separability.

## 5. Conclusions

Data hiding technology in encrypted images was introduced to improve the effectiveness and security of remote sensing image transmission, and a full SRDH-EI algorithm was proposed. The proposed algorithm utilizes compression encoding to reserve space and improve the *EC*, achieving an average *EC* of 2.40 bpp on remote sensing images, enabling efficient transmission, the combination of encryption technology and separable design further enhances the flexibility and security of transmission, and the decrypted image can achieve true full reversibility. However, the algorithm generates a large amount of auxiliary data when reserving space, which affects the *EC*. Further work can consider reducing the amount of auxiliary data for research. In summary, the proposed algorithm provides a technical approach for remote sensing image transmission scenarios with high security, high effectiveness, and separable data reception.

## Figures and Tables

**Table 1:** Changes in image entropy (*α* = 3, *β* = 8, *m* = 0, *T* = 15).

Image	Original Entropy	Entropy after Encryption	Change 1	Entropy after Data Embedding	Change 2	Change 3
Figure 7a	5.40	7.99	2.59	7.98	2.58	0.01
Figure 7b	6.05	7.99	1.94	7.98	1.93	0.01
Figure 7c	6.20	7.99	1.79	7.98	1.78	0.01
Figure 7d	5.93	7.99	2.06	7.98	2.05	0.01
Figure 7e	5.06	7.99	2.93	7.97	2.91	0.02
Figure 7f	7.12	7.99	0.87	7.99	0.87	0.00

**Table 2:** The influence of parameters *m* and *β* on *EC*_1_ in Figure 7a (bpp).

	*m*	1	2	3	4
*β*					
1		0.008	0.015	0.006	0.002
4		0047	0.093	0.070	0.058
8		0.101	0.200	0.160	0.133
12		0.152	0.302	0.252	0.212
16		0.205	**0.407**	0.341	0.281

**Table 3:** Influence of parameter *α* on *EC* (*T* = 1, *β* = 4) for Figure 7a.

Parameter *α*	*EC*/bpp	Decryption Quality/dB	SSIM
1	0.140	*inf*	1
2	0.187	*inf*	1
3	**0.234**	*inf*	1

**Table 4:** Influence of parameter *β* on *EC* (*T* = 1, *α* = 3) for Figure 7a.

Parameter *β*	*EC*/bpp	Decryption Quality/dB	SSIM
1	0.051	*inf*	1
4	0.234	*inf*	1
8	0.479	*inf*	1
12	0.722	*inf*	1
16	**0.969**	*inf*	1

**Table 5:** Influence of parameter *T* on *EC* (*β* = 16, *α* = 3) for Figure 7a.

Parameter *T*	*EC*/bpp	Decryption Quality/dB	SSIM
1	0.969	*inf*	1
3	1.809	*inf*	1
7	**2.234**	*inf*	1
15	1.866	*inf*	1

**Table 6:** Influence of parameter *T* on *EC* (*β* = 16, *α* = 3) for Figure 7b.

Parameter *T*	*EC*/bpp	Decryption Quality/dB	SSIM
1	1.780	*inf*	1
3	**2.** **428**	*inf*	1
7	2.345	*inf*	1
15	1.777	*inf*	1

**Table 7:** Maximum *EC* of Figure 7.

Image	(a)	(b)	(c)	(d)	(e)	(f)	Average
*EC*/bpp	2.23	2.43	2.40	2.24	3.73	1.37	2.40

**Table 8:** Maximum *EC* of Figure 10.

Image	(a)	(b)	(c)	(d)	(e)	(f)	Average
*EC*/bpp	2.22	2.04	1.46	2.26	1.69	0.74	1.74

**Table 9:** Comparison of metrics between the proposed algorithm and related works.

Algorithms	RRBE	Separability	Lossless Decryption	Self-Embedding before Encryption
Bit layer compression. [8]	×	×	×	×
Distributed source encoding. [9]	×	√	×	×
Adaptive prediction error coding. [18]	×	×	×	×
Block classification + prediction. [22]	×	√	×	×
Dynamic multi-layer embedding. [26]	√	√	×	×
Pre-classification + interpolation prediction + error correction. [27]	×	×	×	√
Pre-classification + mapping. [28]	√	√	×	×
MSB prediction + error correction. [30]	√	√	×	×
Interpolation estimation + MSB flipping. [36]	√	×	×	×
The proposed algorithm.	√	√	√	√

## Data Availability

No new data were created or analyzed in this study. Data sharing is not applicable to this article.
